# Change & Grow^®^ Therapeutic Model for Addiction: Preliminary Results of an Interventional Study

**DOI:** 10.3390/bs9120137

**Published:** 2019-12-04

**Authors:** Eduardo Ramadas, Jessica Lopes, Tânia Caetano

**Affiliations:** Department of Research, VillaRamadas International Treatment Centre, 2400-121 Leiria, Portugal; research@villaramadas.com (J.L.); adm@villaramadas.com (T.C.)

**Keywords:** Addiction Treatment, Integrative Treatment, Change & Grow^®^ Model, Depressive Symptomatology, Suicide Ideation, Anxiety, Cognitive Functioning

## Abstract

The last years have seen a paradigm shift concerning addictive disorders, indicating the necessity to study alternative therapeutic models. In this longitudinal study, the objective was to explore the impact of the Change & Grow^®^ therapeutic model developed and used by VillaRamadas on certain psychological variables that frequently appear associated with addiction. A repeated measures (first and last weeks of treatment) design was used, and the psychological measurements were Beck’s Depression Inventory II (BDI-II), Suicide Ideation Questionnaire (SIQ), State-Trait Anxiety Inventory (STAI), and Montreal Cognitive Assessment (MoCA). Results include 26 (16 male and 10 female) patients. Age varied between 17 and 64 years (M = 35.62, SD = 12.60) and duration of treatment between 91 and 193 days (M = 147.35, SD = 27.05). The MoCA total result was significantly higher in the last week of treatment. The results of BDI-II, SIQ, and STAI (both state and trait) were all significantly lower. Neither duration of treatment nor self-reported motivation presented significant correlation values with the difference between measures for any of the variables. The Change & Grow^®^ therapeutic model appears to have an impact on relevant psychological variables in patients admitted into treatment for addictive disorders.

## 1. Introduction

Addiction is widely accepted as one of the growing epidemics of our time. According to the World Drug Report [1], in 2015, 0.6% of the global adult population suffered from some substance use disorder, making up 29.5 million people worldwide. More have problems with harmful use of substances or other addictive disorders not related to substance use. Moreover, the harm caused by substance use is estimated as the loss of 28 million years of “healthy” life globally, attributed to either premature death or disability [1]. 

Despite the recognition of addiction as a global problem and growing efforts to find the best solutions, the complexity of its etiology and development make it challenging to reach that goal. One important factor that adds to the complexity of addiction, is its highly comorbid nature. It has been reported that about 27% of addicts have at least one psychiatric disorder and, of those, 45% have two or more [2]. Some of the psychiatric disorders/complaints most commonly found in comorbidity with addiction are depression [2,3], suicide ideation [4] and anxiety disorders [2,5]. 

Although various psychosocial treatment modalities have research-based evidence for their efficacy [6], there is robust data suggesting that integrated treatment approaches are superior to single-focused treatments [7]. Integrative approaches involve both an interdisciplinary team and the combination of multiple evidence-based treatments [7]. These two factors facilitate the fulfillment of the principles for effective treatment created by the National Institute on Drug Abuse (NIDA; [8]), such as the necessity to attend to the multiple needs of patients, including comorbid complaints. 

Unlike other disorders, the complexity of addiction and its treatment make unrealistic the global adoption of manualized evidence-based protocols [9]. Instead, broad therapeutic principles can be useful in guiding the creation of effective treatment approaches that take into account the wider context. In a review of public guidelines, Glasner-Edwards and Rawson [9] indicate four main evidence-based principles for psychosocial intervention in addiction: 1) contingency management principles (e.g., Community Reinforcement Approach; [10]); 2) motivational interviewing techniques [11]; 3) cognitive behavioral coping skills and relapse prevention strategies [12]; and 4) couples/family counseling techniques [13]. 

Nevertheless, while integrative treatment approaches seem to be the most successful, there is more to recovery than formal treatment. Studies [14] indicate that for both short-term and long-term recovery, factors such as consequences of addiction (translating into motivation for abstinence), social support and affiliation to 12-step programs are essential.

Taking into consideration the factors presented above, a new model of intervention was created to be used in the context of an intensive in-patient treatment. The Change & Grow^®^ Model combines techniques from all the major evidence-based therapeutic approaches while also providing an underlining treatment structure, which takes into account the timing of the introduction of each of these techniques. Besides the integration of different evidence-based principles, the model also adopts several concepts of the 12-steps program and a complete abstinence approach.

While the long-term goal is the study of the efficacy of the model regarding the recovery from addiction as a whole, this first preliminary study looks into its possible impact on comorbid complaints. The objective was to explore the impact of the treatment model on depressive symptomatology, anxiety and suicide ideation, all psychological complaints frequently associated with addiction. 

## 2. Materials and Methods

### 2.1. Participants

The sample used in this study was composed of 26 (16 male and 10 female) Portuguese patients who initiated and successfully completed treatment at VillaRamadas International Treatment Centre throughout 2018. Age varied between 17 and 64 years (M = 35.62, SD = 12.60) and duration of treatment between 91 and 193 days (M = 147.35, SD = 27.05). 

Inclusion criteria were age of 16 or above, proficiency of the Portuguese language, the presence of an addictive disorder (diagnosed by an in-house psychiatrist), successful completion of the therapeutic program. 

Some of the participants had a dual diagnosis (mainly anxiety, affective and personality disorders), but the choice was made not to control for this variable because of the small sample size. Similarly, most of the patients received tailored pharmacological treatment throughout the program, which was impossible to effectively control because of its individualized and dynamic approach (i.e., medication was adjusted as needed by the in-house psychiatrist). There was no standardized pharmacological protocol, consequence of the high heterogeneity of our sample regarding psychiatric complaints. 

### 2.2. Evaluation Procedure

As part of the normal intake procedure at VillaRamadas, patients are evaluated by an in-house psychiatrist and administered an evaluation protocol in which are included the measures used in this study.

The evaluation protocol has been computerized into a proprietary online platform that can only be accessed by the certified clinical psychologists who integrate the therapeutic staff of the center. Upon the admission of a new patient, a patient code is generated, which allows for the creation of an individual patient page on the aforementioned evaluation online platform. It is during the second consultation (second day in the treatment center) with an assigned psychologist that the patient is first exposed to this evaluation page (T0), which opens with an explanation of the importance of psychometric evaluation and a declaration of informed consent. The psychologist explains that the patient can refuse the administration of the measures and the use of the results for research purposes without any negative repercussions. If preferred, the tests can also be administered in paper format. 

The second, and for now last, moment of evaluation happens during the last week in treatment (T1). The same conditions of administration apply. 

### 2.3. Treatment Procedure—Change & Grow^®^ Model

The Change & Grow^®^ Model is an integrative therapeutic model developed throughout the last ten years at VillaRamadas International Treatment Centre with the objective of adapting existing programs and useful psychological techniques into one integrative approach for addictive disorders. 

Conceptually, the model considers relevant theoretical and practical knowledge from cognitive and behavioral therapy, third generation therapies, positive psychology, motivational interviewing, the 12-step program, and community reinforcement approach. The model also advocates a complete abstinence approach.

Structurally, the model comprises five different therapeutic stages, or principles, with distinct therapeutic goals. The principles are Truth, Acceptance, Gratitude, Love and Responsibility. There are necessary therapeutic activities for each stage and at its completion, a colored bracelet is given to the patient in representation of their treatment progress and as positive reinforcement. 

The first principle, Truth, focuses on the past, namely the patient’s life story, resentments, damages done to self or others (negative consequences of use) and the processing of negative emotions like guilt and shame. This first stage is essential for promoting motivation both for the treatment itself and for recovery and abstinence in geral. The second, Acceptance, introduces the third generation concept of accepting emotions and thoughts while trying to promote a change of behavior. The third, Gratitude, intends to practice a change of perspective from the negative to the positive. The fourth principle, Love, focuses on self-compassion and related concepts as important self-regulatory strategies. Lastly, Responsibility promotes autonomy and self-efficacy, and more practical skills such as planning, goal-setting, and relapse prevention. 

The presented stages/principles are part of an individual treatment process and at any given moment different patients are situated in different stages. However, the program gives special importance to the group and, as such, a considerable part of the therapeutic schedule is spent on various group sessions (e.g., psychoeducation, sharing groups, creative therapy).

During each weekday, patients have four group sessions (two of 90 minutes and two of 60 minutes, intercalated) mediated by either a psychologist or a social worker, one group session to end the therapeutic day, and one individual session with a certified and trained clinical psychologist. During the weekend, patients have two group sessions (opening and ending of the day) and no individual sessions and have an escorted (by members of the therapeutic staff) visit outside the facility (e.g., beach, museum, sports). Regarding exercise, two times per week patients have the option to have 90 minutes of guided exercise with a personal trainer, and can otherwise access the gym outside the therapeutic schedule (every day after 17:00). 

In the final stage of treatment, patients start to attend aftercare sessions (structured similarly to 12-step groups) provided by the centre. 

Throughout the treatment, families are in contact with the primary therapist and participate in a number of family therapy sessions scheduled according with the patient’s progress. 

More information about the Change & Grow^®^ model can be found as supplementary material. 

### 2.4. Ethics Statement 

No formal ethical review was necessary to conduct the present study since all the procedures were those regularly used in the treatment center and no changes needed to be made. Nonetheless, the regular ethical considerations were assured with the informed consent given by the patients and the necessary anonymization of personal and clinical data.

### 2.5. Measures

The following measures were chosen taking into consideration their availability in the Portuguese language as ease and time of administration.

1. Sociodemographic data: during intake, the patients completed a sociodemographic and clinical history questionnaire. The relevant data used in this study were gender, age, and years of education.
2. Motivation: a simple self-report question was used to screen for motivation. The question was “How motivated for treatment do you consider yourself to be?” The question was scored on a 5-point Likert scale from 0 (“Not at all”) to 4 (“Very”).
3. Duration of Treatment: measured in days and calculated automatically upon the completion of treatment. At admission, standard treatment duration is chosen (90, 135 or 180 days) depending on the idiosyncrasies of each patient (diagnosis, clinical history, the existence of previous treatments, etc.). The date of completion can be changed by the therapeutic team if they consider the patient is not ready.
4. Depressive Symptomatology: measured with the Beck Depression Inventory II (BDI-II; [15,16]), which is one of the most commonly used instruments both in research and practice to assess the presence and severity of depression. The inventory is composed of 21 sets of statements assessing symptoms corresponding to criteria for the diagnosis of the depressive disorders listed in the DSM-IV [17]. The answer options include four levels of severity with scores for each item ranging from 0 to 3. The total score is the sum of all responses and can vary from 0 to 63, with higher scores representing more depressive symptomatology. In the current study, BDI-II presented a Cronbach’s alpha of 0.947 for the first measure, and of 0.853 for the second. All the participants completed the BDI-II at both moments of evaluation.
5. Suicide Ideation: measured using the Suicide Ideation Questionnaire (SIQ; [18,19]), which is a self-report instrument composed of 30 items scored in a 7-point Likert scale from 0 (“Never had this thought”) to 6 (“Almost every day”). The total score can vary from 0 to 180, with higher scores indicating more frequent suicide-related cognitions. Reynolds [18] considers a score of 41 or higher to be potentially indicative of significant psychopathology and suicide risk. In the present study, SIQ showed a Cronbach’s alpha of 0.973 for the first measure, and of 0.959. Twenty-five participants completed the SIQ at both moments of evaluation.
6. Anxiety: measured with the State-Trait Anxiety Inventory (STAI; [20,21]), a self-report instrument composed of two scales of 20 items each, which evaluate state (Y1) and trait (Y2) anxiety respectively. Items are scored on a 4-point Likert scale from 1 (“No/Almost Never”) to 4 (“Very/Almost Always”), and the total score for each scale can vary from 20 to 80. Higher scores indicate higher levels of (state or trait) anxiety. Spielberger considered scores of 47 (for state-anxiety) and 42 (for trait-anxiety) as cut-off points to define the possible existence of pathological levels of anxiety. In the current study, STAI-Y1 and STAI-Y2 presented Cronbach’s alphas of 0.941 and 0.958 for the first measure, and of 0.932 and 0.948 for the second. Twenty-five participants completed the STAI at both moments of evaluation.
7. Cognitive Functioning: measured with the Montreal Cognitive Assessment (MoCA; [22,23]) was used. MoCA is composed of an evaluation protocol of a single page that considers the following cognitive domains: memory (deferred evocation of words), visuospatial capacity (clock drawing and cube copy), executive function (trail making test B, phonemic verbal fluency, and verbal abstraction), language (nomination of three animals, repetition of two syntactically complex phrases and phonemic verbal fluency), orientation (temporal and spatial), attention, concentration and working memory (digits memory, sustained attention task and serial subtraction of 7). For this study, merely the MoCA total score is used. Only nine participants completed the MoCA at both moments of evaluation.

### 2.6. Data Analysis

IBM SPSS version 23 statistical package was used for data analysis. 

Reliability analyses were conducted for every measure used and for both moments of evaluation.

There were computed new double variables of the differences between measures, with positive and negative differences to facilitate the interpretation in distinct analyses. For the negative values of all the variables but MoCA, the values were computed by subtracting the values for the first measure from the values for the second measure. The reverse was done to compute the positive values. 

Descriptive statistics were used to study the characteristics of the sample.

Kurtosis and skewness values for the differences between moments of evaluation were observed to determine the normality of the data. Despite the confirmation of normality, the small sample size justified the choice of non-parametric alternatives for subsequent analyses. 

Spearman correlation values were used to explore the relationships between the differences between measures and the other both age, years of education, self-reported motivation, duration of treatment and baseline measures.

Finally, the Wilcoxon signed ranks test was used to study the possible existence of differences between moments of evaluation. 

## 3. Results

### 3.1. Descriptive Statistics

#### 3.1.1. Sociodemographic Characterization of the Sample and Variables’ Means at Both Measures

Presented in Table 1 are the descriptive statistics for the relevant sociodemographic data and for the studied variables at both moments of measure (first and last week of treatment). Regarding the participants who completed both measures of MoCA, age ranged from 18 to 45, and total result from 18 to 28 and 25 to 28 (for first and second measures respectively). 

#### 3.1.2. Descriptive Statistics for the Differences between Measures

Negative difference values were used for all the variables but MoCA, for which positive values were preferred. 

In Table 2 are presented the descriptive statistics for the computed variables of the differences between the two measures for each variable in study. 

### 3.2. Spearman Correlation

To facilitate interpretation, the positive difference values of every variable were used for the Spearman correlation analyses.

There were no significant correlation values between the differences between measures of the psychological variables and age, years of education, and duration of treatment. There was a significant moderate negative association between self-reported motivation and the difference in SIQ results between measures. 

As expected, the differences between measures presented strong or very strong positive associations with base-lines measures of the same variable. These results indicate that higher baseline measures are associated with a bigger difference between measures for every psychological variable but the MoCA total score. For MoCA, lower base-line measures associate with bigger differences between measures. 

The full results are presented in Table 3. 

### 3.3. Wilcoxon Signed Ranks Test

According to the Wilcoxon signed ranks test, scores on BDI-II, SIQ, STAI-Y1, and STAI-Y2 were significantly lower on the second measure, done at the last week of treatment, in comparison to the first measure at the first week. Scores on MoCA were significantly higher. 

The summary of the test’s results can be found in Table 4. 

## 4. Discussion

While there are various integrative models for the treatment of addiction, to our knowledge there is none that both incorporates all the major psychological approaches that have been proven effective and offers a structure that takes into consideration the timing of their introduction. We believe that this structure provides an opportunity to maximize the therapeutic gains by introducing each approach in the right moment of treatment.

Our long-term goal is to study the efficacy of the model in promoting recovery and positively impacting psychopathological complaints that appear often associated with addictive disorders (e.g., depressive symptomatology, anxiety). The present preliminary study focused on this second objective.

The results indicate that all the studied variables suffered significant changes between the two moments of evaluation (beginning and end of treatment). Depressive symptomatology, suicide ideation, state, and trait anxiety reduced significantly from the first week of treatment to the last, while general cognitive functioning increased as expected. 

These data suggest that the Change & Grow^®^ Model could be effective in addressing these complaints, which frequently appear in comorbidity with addiction. Its possible efficacy is in line with current literature regarding addiction and dual disorders treatment. Research suggests that a highly structured and intensive treatment program [24], which adopts an integrative approach [7,24], can effectively treat both minor and serious comorbid complaints. 

Besides suicide ideation, of which the difference between measures presented a significant moderate positive association with self-reported motivation, no other variables difference values showed any association with years of education, self-reported motivation or duration of treatment. These results, although preliminary and based on a small sample, could indicate that the treatment impact on the studied psychological variables is not moderated by level of education or duration of treatment.

The literature relating to the considered possible moderators differs. While voluntary admission into treatment is not seen as a requirement for treatment success [8], motivation has been associated with positive outcomes [14]. The moment of admission therefore, may not be the best moment to measure self-reported motivation, if we pretend to study its impact. 

Regarding duration of treatment, NIDA guidelines [8] indicate that duration has an impact on its success and establishes a three months minimum. The lack of moderation could be due to the fact that the patients included in the sample had all completed programs which respected that minimum (90, 135 or 180 days). 

Finally, although level of education did not present itself as a moderator in this study, it remains an important variable to consider regarding treatment success. Collected in the context of a private treatment center, our sample is skewed towards higher levels of education. 

Despite the positive results, the study presents significant limitations that we hope to overcome in future research endeavors, such as a small sample size, the lack of a control group, and no post-treatment follow-up. 

The small sample size limits the data analyses that can be conducted, making it impossible to test the possible moderator effects of important control variables such as level of education, duration of treatment and self-reported motivation. It would also be important in future research, when this limitation is no longer present, to understand the differential impact of the treatment program according to gender, type of addiction, as well as other relevant variables. Moreover, this limitation is especially relevant for the results concerning the changes in cognitive functioning, since only nine participants completed the chosen measure (MoCA) on both moments of evaluation.

The lack of control group, a consequence of the contextual limitations of the treatment center, makes it impossible to understand how the presented model and treatment program compares to other evidence-based treatment approaches, both single focused and integrative ones. 

Lastly, the absence of a post-treatment follow-up measure does not allow for the study of the maintenance of the therapeutic gains, or how these impact sobriety or relapse. We hope to eliminate this limitation in future research.

In line with the recent shift of focus into process-based therapy, we hope that by addressing the limitations presented above we will be capable of studying not only the impact of treatment but also the process by which this impact is achieved. It would be important to understand which variables mediate the therapeutic gains and if those variables differ with the phase of treatment. 

## 5. Conclusions

We believe that although the current study only presents preliminary results and has, as stated above, significant limitations, the results suggest that the model/treatment program successfully impacts various psychological complaints that frequently appear in comorbidity with addictive disorders. We hope that this first presentation of the model will generate interest from researchers and clinicians alike and promote discussion about the state of the art regarding integrative treatments for addiction. Future research should provide a more in-depth look at the structure of such programs and the timing of the introduction of different techniques and approaches. 

## Figures and Tables

**Table 1:** Descriptive Statistics for sociodemographic data and variables at both measures.

Variables	*N*	Minimum	Maximum	*M*	*SD*
Age	26	17	64	35.62	12.60
Years of Education	26	4	17	12.69	2.71
Duration of Treatment	26	91	193	147.35	27.05
Motivation Level	26	1	4	3.46	0.81
BDI-II—1st measure	26	3	52	17.31	13.45
BDI-II—2nd measure	26	0	21	5.23	4.89
SIQ—1st measure	26	0	134	46.69	47.22
SIQ—2nd measure	25	0	91	19.08	27.16
STAI-Y1—1st measure	26	22	74	44.54	13.07
STAI-Y1—2nd measure	25	23	64	34.80	9.79
STAI-Y2—1st measure	26	22	80	52.04	14.63
STAI-Y2—2nd measure	25	22	58	36.80	11.21
MoCA—1st measure	21	18	29	24.90	2.51
MoCA—2nd measure	13	25	30	27.46	1.34

*Note.* n = sample size; M = mean; SD = Standard Deviation.

**Table 2:** Descriptive Statistics for the differences between measures.

Variables	*N*	Minimum	Maximum	M	SD
Difference BDI-II	26	−41	0	−12.08	12.00
Difference SIQ	25	−126	3	−29.48	38.75
Difference STAI-Y1	25	−29	9	−10.24	10.20
Difference STAI-Y2	25	−39	6	−15.76	11.30
Difference MoCA	9	0	7	2.56	2.19

*Note*. n = sample size; M = mean; SD = Standard Deviation.

**Table 3:** Spearman correlation values between the studied variables.

Variables	1	2	3	4	5	6	7	8	9	10	11	12	13	14
1. Age	-													
2. Years of Education	0.202	-												
3. Motivation	0.391*	0.249	-											
4. Duration of Treatment	−0.053	0.294	−0.113	-										
Base-line measures														
5. BDI-II	−0.225	0.046	−0.297	0.032	-									
6. SIQ	−0.113	0.201	−0.140	−0.161	0.777**	-								
7. STAI-Y1	−0.126	0.137	−0.365	−0.055	0.808**	0.759**	-							
8. STAI-Y2	0.163	0.212	0.042	−0.162	0.601**	0.844**	0.621**	-						
9. MoCA	0.070	0.011	0.017	−0.035	−0.385	−0.322	−0.108	−0.086	-					
Differences between measures														
10. BDI-II	−0.188	0.050	−0.332	0.229	0.820**	0.608**	0.606**	0.549**	−0.511*	-				
11. SIQ	−0.286	0.016	−0.412*	0.044	0.672**	0.758**	0.667**	0.570**	−0.299	0.700**	-			
12. STAI-Y1	−0.079	0.306	−0.221	0.222	0.618**	0.469*	0.716**	0.454*	−0.448*	0.739**	0.512**	-		
13. STAI-Y2	−0.039	0.313	−0.038	0.113	0.452*	0.480*	0.393	0.661**	−0.074	0.646**	0.443*	0.591**	-	
14. MoCA	−0.332	0.242	−0.044	0.640	−0.064	−0.255	−0.506	−0.553	−0.922**	0.506	0.102	0.494	0.111	-

*Note*. Figures in **bold** are significant correlation values. Significance level = * *p* ˂0.05, ** *p* ˂0.01.

**Table 4:** Summary of Wilcoxon signed ranks test results.

Differences	Negative Ranks			Positive Ranks			Test Statistics		
	*n*	Mean Rank	Sum of Ranks	*n*	Mean Rank	Sum of Ranks	Ties	*Z*	*p*
(BDI-II 2nd)—(BDI-II 1st)	25	13.00	325.0	0	0.00	0.0	1	−4.377 ^a^	0.000
(SIQ 2nd)—(SIQ 1st)	18	14.50	261.0	5	3.00	15.0	2	−3.742 ^a^	0.000
(STAI-Y1 2nd)—(STAI-Y1 1st)	20	14.75	295.0	5	6.00	30.0	0	−3.567 ^a^	0.000
(STAI-Y2 2nd)—(STAI-Y2 1st)	22	14.39	316.5	3	2.83	8.50	0	−4.145 ^a^	0.000
(MoCA 2nd)—(MoCA 1st)	0	0.00	0.0	7	4.00	28.0	2	−2.384 ^b^	0.017

*Note. n* = sample size; *Z* = Wilcoxon signed ranks test; ^a^ Based on negative ranks; ^b^ Based on positive ranks; *p* = significance level.

## Supplementary Materials

The following are available online at https://data.mendeley.com/datasets/gj9gmnymxk/1: Raw data: Dataset_Article, Data analysis output: Output_Article_Results, Change & Grow^®^ principles, steps and promises: Change & Grow Model.
